# ﻿Two new species and records of *Neoperla* (Plecoptera, Perlidae) from Yunnan, China

**DOI:** 10.3897/zookeys.1092.78069

**Published:** 2022-04-04

**Authors:** Yingying Wang, Wenliang Li, Weihai Li

**Affiliations:** 1 College of Horticulture and Plant Protection, Henan University of Science and Technology, Luoyang, Henan 471023, China Henan University of Science and Technology Luoyang China; 2 Department of Plant Protection, Henan Institute of Science and Technology, Xinxiang, Henan 453003, China Henan Institute of Science and Technology Xinxiang China

**Keywords:** New record, new species, Perlinae, stoneflies, Yunnan

## Abstract

Two new species of the stonefly genus *Neoperla*, *N.gaoligongshana***sp. nov.** and *N.hajeki***sp. nov.** are described from Yunnan Province of southwestern China based on the morphological and distributional data, and the new species are compared with two congeners, *N.wuzhishana* Chen & Du, 2016 and *N.orissa* Stark & Sivec, 2015. *Neoperlahubleyi* Stark & Sivec, 2008 is recorded from Yunnan Province for the first time.

## ﻿Introduction

*Neoperla* Needham, 1905 is the largest genus in Perlinae, with more than 270 species worldwide. In China, there are at least 110 valid species described by Chen and Du (2015, 2016a, 2016b, 2016c, 2017, 2018), Chu (1929), Claassen (1940), Dewalt et al. (2021), Du (1998, 1999, 2000a, 2000b), Du et al. (1999, 2001), Du and Sivec (2004, 2005), Du and Wang (2005, 2007), Huo et al. (2021), Kong et al. (2014), Kong and Li (2016), Li et al. (2011a, 2011b, 2012a, 2012b, 2013a, 2013b, 2014a, 2014b, 2017, 2019, 2020), Li et al. (2021), Li and Li (2013a, 2013b), Li and Liu (2019), Li and Wang (2011), Li and Zhang (2014), Mo et al. (2019, 2020a, 2020b, 2021a, 2021b), Murányi et al. (2015), Needham (1905), Qin et al. (2013), Sivec et al. (1988), Sivec and Zwick (1987), Stark and Sivec (2008, 2015), Wang et al. (2013a, 2013b, 2014), Wu (1935, 1938, 1948, 1962, 1973), Wu and Claassen (1934), Yang et al. (2017), and Yang and Yang (1990, 1991, 1992, 1993, 1995a, 1995b, 1996, 1998), Yang and Li (2018), and Zwick (1973, 1977, 1983, 1986). Eight species of *Neoperla* are known from Yunnan Province: *N.cavaleriei* Navás, 1922, *N.diehli* Sivec, 1985, *N.lihuae* Li & Murányi, 2014, *N.limbatella* Navás, 1933, *N.lui* Du, 2004, *N.yanlii* Li & Wang, 2014, *N.yunnana* Li & Wang, 2014, and *N.obscurofulva* Wu, 1962. Yunnan Province is located in southwestern China. The region is adjacent to Guizhou and Guangxi in the east, Tibet to the northwest, Myanmar to the west, Sichuan to the north, and Laos and Vietnam to the south. The total area of Yunnan Province is 3941 million square kilometers.

We recently examined a collection of stonefly specimens received from the National Museum Prague of the Czech Republic, and some of the results of our investigations into this material have been already published (Mo et al. 2019, 2020b, 2021a, 2021b; Li and Kong 2020; Li et al. 2019, 2020). Herein, we report our results on the *Neoperla* from Yunnan Province in this collection, including two new species and one new record from the province.

## ﻿Materials and methods

Types are kept in the Insect Collection of Henan Institute of Science and Technology (**HIST**), Xinxiang of China and the National Museum Prague (**NMP**) of the Czech Republic, and the Collection of Smaller Insect Orders, Department of Zoology, Hungarian Natural History Museum (**HNHM**), Budapest, as indicated in the text. Specimens used in this study were collected using a light trap and stored in 75% ethanol. Specimens were examined with the aid of a Leica M420 dissecting microscope and the color photographs were taken with a Leica S8APO stereo microscope. Aedeagi were everted using the cold-maceration technique of Zwick (1983). The morphological terminology follows that of Stark and Sivec (2008). The map (Fig. 7) was prepared using a base map of Yunnan Province downloaded from DataV.GeoAtlas (Alibaba, China) and ACME Mapper 2.2 (http://datav.aliyun.com/tools/atlas/index.html; http://mapper.acme.com/).

## ﻿Results and discussion

### ﻿ Neoperla gaoligongshana sp. nov.

Taxon classificationAnimaliaPlecopteraPerlidae

ED5F6033-5644-50B2-84E8-FE9113683BDB

http://zoobank.org/69D93D93-861F-4F64-98BC-0F01F862F255

Figs 1 , 2

#### Material examined.

***Holotype***: male (NMP), China: Yunnan Province, Baoshan City, Gaoligongshan National Nature Reserve, Baihualing Village, 6–8.VII.2016, 25°17.7'N, 98°48.1'E, 1535 m, light trap, leg. J. Hájek & J. Růžička.

#### Diagnosis.

Males of this species are characterized by having the tergum 7 with a triangular process with sparse spines apically. The aedeagal tube is straight, and aedeagal sac is armed with spines.

#### Description.

**Figure 1.**
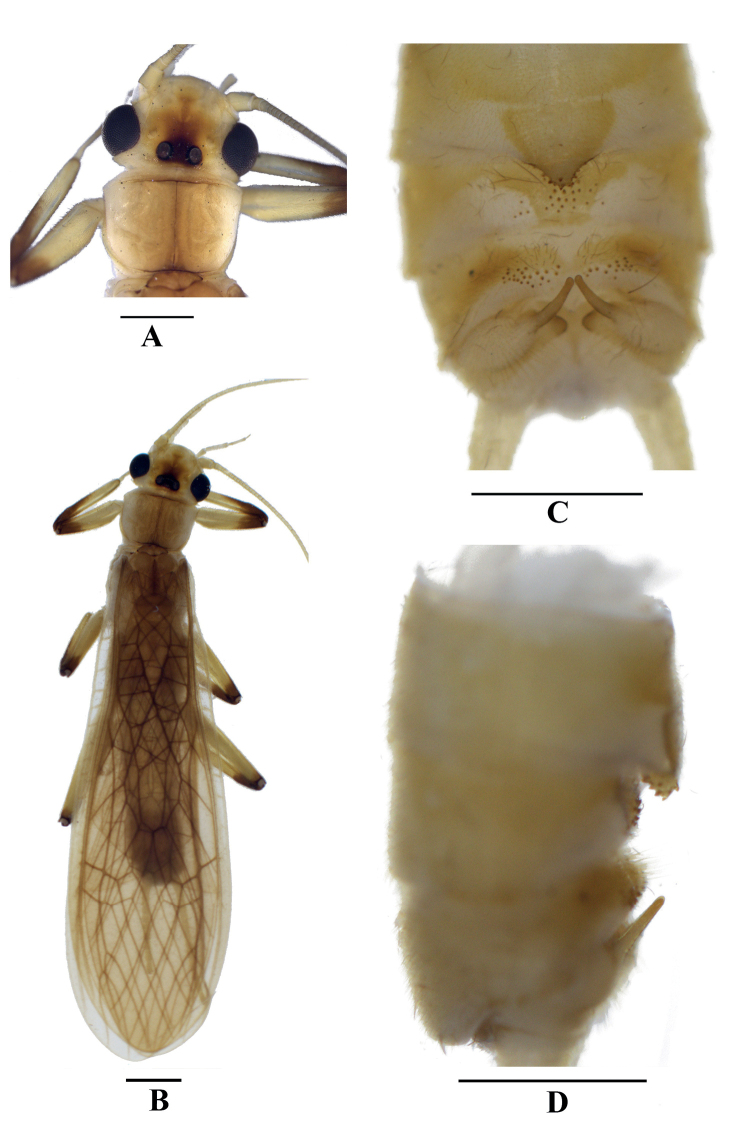
*Neoperlagaoligongshana* sp. nov. (male) **A** head and pronotum, dorsal view **B** adult habitus, dorsal view **C** terminalia, dorsal view **D** terminalia, lateral view. Scale bars: 1 mm.

Adult habitus (Fig. 1). Body color brown. Head mostly yellowish brown, with a black marking covering ocellar triangle, the marking extended forward to pale M-line and getting brown, and a narrow triangular marking occurring forward of M-line. Head approximately as wide as the pronotum; compound eyes black; antenna and palpi yellow. Distance between ocelli slightly wider than the diameter of an ocellus. Pronotum disc brown, midline darker, margins pale (Fig. 1B, C). Wings subhyaline, veins brown; legs pale to yellowish brown, femorotibial joint dark brown. Abdomen brownish, cerci brownish (Fig. 1B, C).

**Figure 2.**
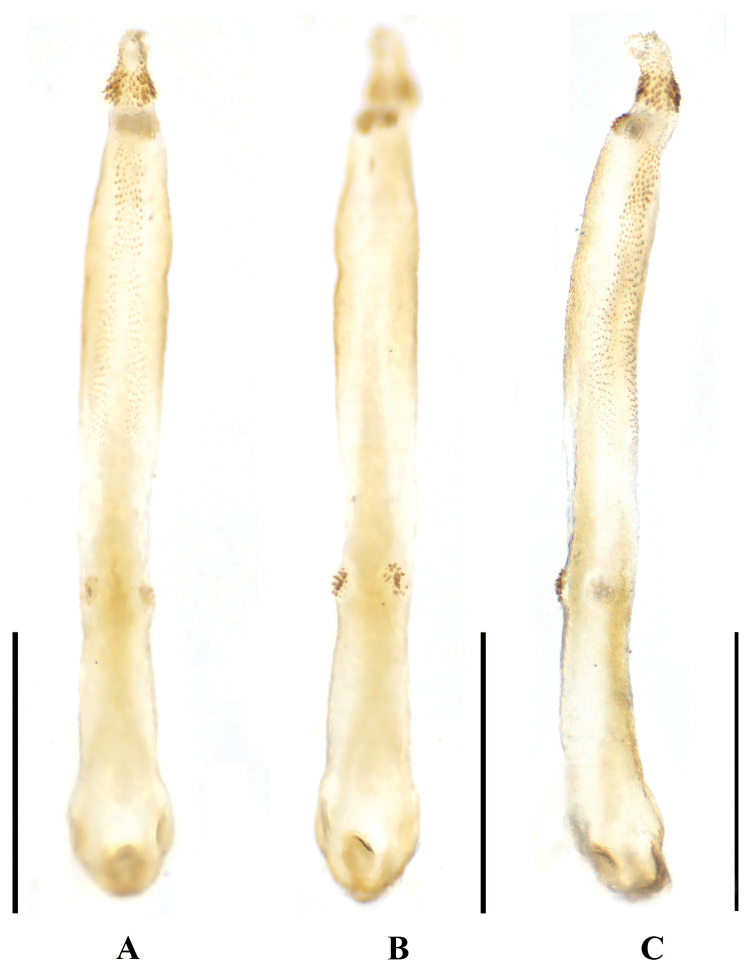
*Neoperlagaoligongshana* sp. nov. (male) **A** aedeagus with everted sac, dorsal view **B** aedeagus with everted sac, ventral view **C** aedeagus with everted sac, dorsolateral view. Scale bars: 1 mm.

**Male** (Figs 1, 2). Forewing length ca 10.9 mm, hindwing length ca 9.7 mm. Tergum 6 unmodified. Process of tergum 7 triangular, apex rounded and margined by sparse spines (Fig. 1B, D). Tergum 8 with a median sensilla basiconica patch on a trapezoidal sclerite. Tergum 9 with two lateral patches of several sensilla basiconica and long hairs. Hemitergal processes slender, straight in lateral aspect, apex obtuse (Fig. 1B, D).

***Aedeagus*** (Fig. 2). Aedeagal tube weakly sclerotized ventrally and dorsally sclerotized strongly, apex with two ventral spinous lobes (Fig. 2A, B). Sac nearly straight, ca 2× as long as the tube. Spinose apex of sac slender, slightly ventrally curved, with an apical dorsolateral patch of black spines and a subapical ventral patch of spines (Fig. 2A, C); two wide rows of numerous smaller spines covering most of the dorsal surface of the sac, basal half of spinous rows interrupted medially (Fig. 2A, C).

**Female.** Unknown.

#### Etymology.

The specific name refers to the Gaoligongshan National Nature Reserve in Yunnan Province, where the type locality is situated.

#### Distribution.

China (Yunnan).

#### Ecology.

Gaoligongshan National Nature Reserve is located in northwestern Yunnan Province and is the largest nature reserve of the province. At the same locality, accompanying stoneflies were *Neoperlahajeki* sp. nov. and *Tyloperlailliesi* Stark & Sivec, 2005.

#### Remarks.

The new species is a member of the *N.montivaga* group. The aedeagal tube and terga 7–10 of the new species are similar to those of *Neoperlawuzhishana* Chen & Du, 2016, but *N.wuzhishana* can be distinguished from the new species primarily by the shape of the aedeagal sac and aedeagal armatures (Fig. 8C). In *N.wuzhishana*, the aedeagal sac is distinctly curved and expanded apically (Fig. 8C, present study) (which is obscure in the original drawing because of an apical damage in the type) and has at least four large dorsal spines subapically (see figs 7, 8 in Chen, Du 2016b); however, the aedeagal sac of *N.gaoligongshana* is nearly straight and the apex is constricted and has only small spines subapically on its dorsal side. In addition, the aedeagal sac of *N.wuzhishana* bears a dozen moderately long spines at mid-length which are absent in *N.gaoligongshana*. Besides, the color pattern including that of the head and legs of *N.wuzhishana* and *N.gaoligongshana* is different: *N.wuzhishana* has brown legs and the head is pale with a small dark spot between the posterior ocelli, while in *N.gaoligongshana*, the legs are pale to yellowish brown, the femorotibial joint is dark brown, and the head has a large, black marking covering the ocellar triangle, with this marking extending anterolaterally under the pale M-line (Fig. 1B).

We examined specimens of *N.wuzhishana* from Yinggeling, Hainan Province, and found slight intraspecific variations of head pattern and armatures of the aedeagal sac: the dark area between ocelli in males was slightly smaller than in females, which in both sexes are quite small (Fig. 8A, B); four large spines in types (both in the original illustrations and descriptions) (see figs 7, 8 in Chen, Du 2016b), which can number seven to nine in our specimens (Fig. 8C, D). Therefore, the absence of large aedeagal spines in *N.gaoligongshana* is regarded as a distinguishing character, separating it from *N.wuzhishana*.

### ﻿ Neoperla hajeki sp. nov.

Taxon classificationAnimaliaPlecopteraPerlidae

3377522A-EAE5-5D1C-9A50-A2E6AC16C790

http://zoobank.org/2E0FD484-BE6F-4350-9C62-30AB5B651779

Figs 3 , 4 , 5

#### Type material.

***Holotype***: male (NMP), China: Yunnan Province, Baoshan City, Zizhi Village, 29.VI–2.VII.2016, 25°43.7'N, 98°34.1'E, 1995 m, light trap, leg. J. Hájek and J. Růžička. ***Paratypes***: 9 females (NMP), 1 male and 3 females (HIST), 1 male and 2 females (HNHM), same date as holotype.

#### Diagnosis.

This species is characterized by a small dark marking over the ocellar area and a dark brown stigma before the M-line. The male of new species is characterized by an S-shaped aedeagal tube and by a sac bearing a subapical triangular patch of spinules in dorsal aspect.

#### Description.

**Figure 3.**
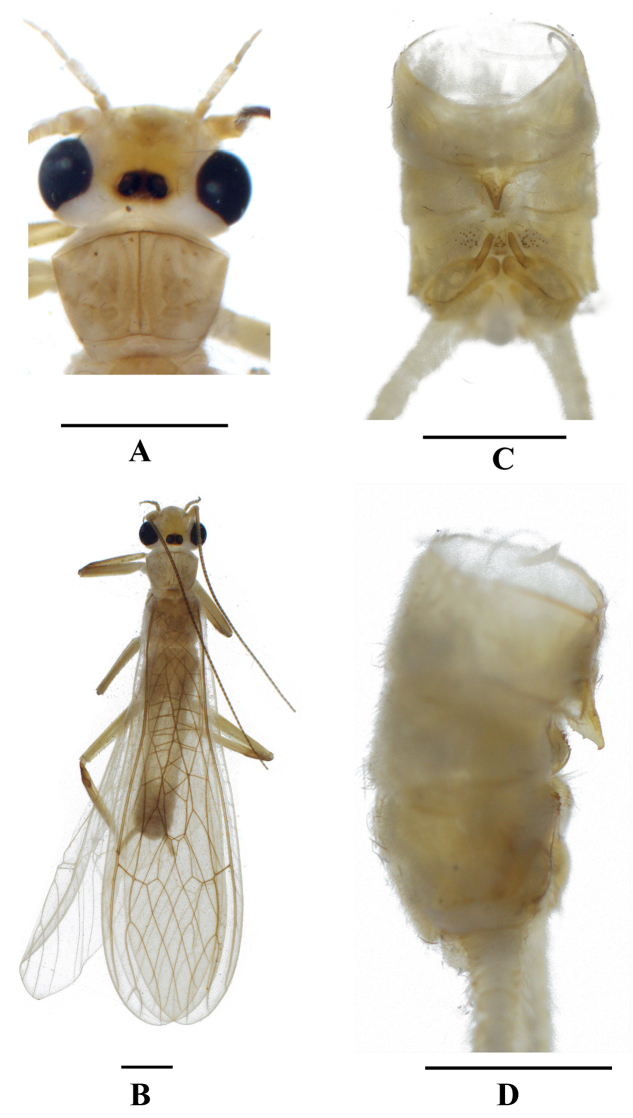
*Neoperlahajeki* sp. nov. (male) **A** head and pronotum, dorsal view **B** adult habitus, dorsal view **C** terminalia, dorsal view **D** terminalia, lateral view. Scale bars: 1 mm.

Adult habitus (Figs 3, 5). General body color brown. Head general pale brown, a small dark marking covers ocellar area, with a dark brown stigma before M-line. Head slightly wider than pronotum; compound eyes blackish, antenna dark brown except the basal segment yellowish (Figs 3A, 5A), palpi pale brown. Distance between ocelli narrower than the diameter of ocellus and a small marking between ocelli dark brown. Pronotum yellow, with rugosities and a strip-like midline (Figs 3A, 5A). Wings hyaline, veins brown; legs brown, basal part of femur dark brown, with wider yellow bands in mid- and hind legs (Figs 3B, 5B). Cerci yellowish (Figs 3D, 5B).

**Figure 4.**
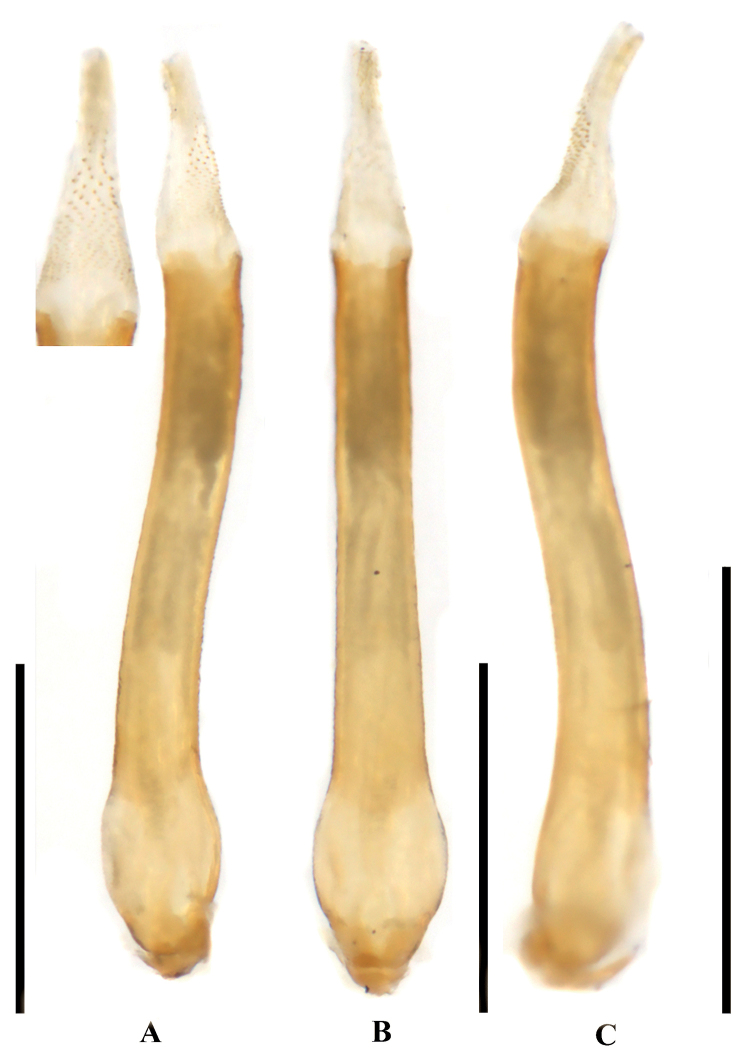
*Neoperlahajeki* sp. nov. (male) **A** aedeagus with everted sac, dorsal view **B** aedeagus with everted sac, ventral view **C** aedeagus with everted sac, lateral view. Scale bars: 1 mm.

**Male** (Figs 3, 4). Forewing length 9.1–9.3 mm, hind wing length 7.9–8.1 mm. Process of tergum 7 sclerotized and triangular, with a nipple-like apex and covered with small sensilla basiconica (Fig. 3B, D). Tergum 8 with a median plump mound and a few sensilla basiconica, mostly covered by the process of tergum 7. Tergum 9 with two separated mesal projections covered by sensilla basiconica and hairs. Hemitergal processes of tergum 10 finger-like in shape, slightly curved medially in dorsal aspect, apex acute (Fig. 3B, D).

***Aedeagus*.** Aedeagal tube sclerotized, slightly S-shaped in lateral aspect. Sac short, ca 1/3 as long as the tube (Fig. 4). Aedeagal sac bearing a subapical triangular patch of spinules in dorsal aspect (Fig. 4A), remainder bald except a few ventral spinules (Fig. 4B, C).

**Figure 5.**
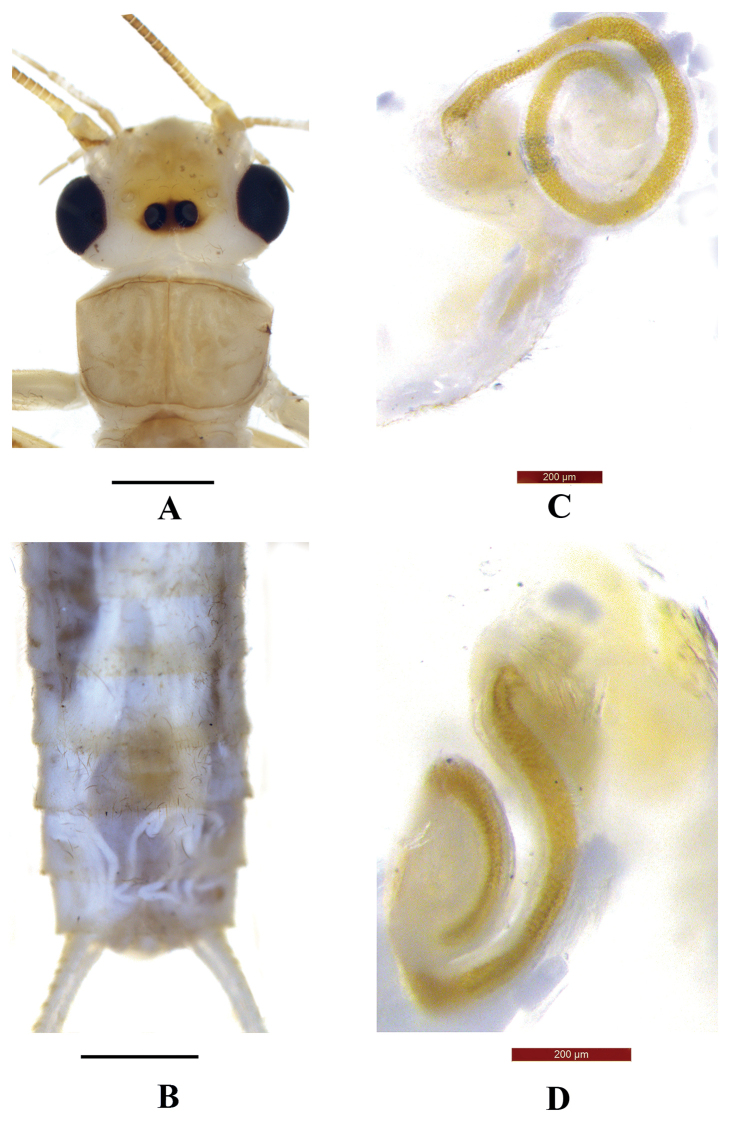
*Neoperlahajeki* sp. nov. (male) **A** head and pronotum, dorsal view **B** terminalia, dorsal view **C** vagina, dorsal view **D** vagina, lateral view. Scale bars: 1 mm (**A–B**); 0.2 mm (**C–D**).

**Female** (Fig. 5). Forewing length 9.3–9.6 mm, hind wing length 8.2–8.4 mm. General color pattern is similar to males. The subgential plate of sternum 8 is not produced posteriorly. Vagina large and apically slender, spiral and incurved, apical round, full of scaly spots. Spermatheca small, its origin in the terminal vagina.

**Figure 6.**
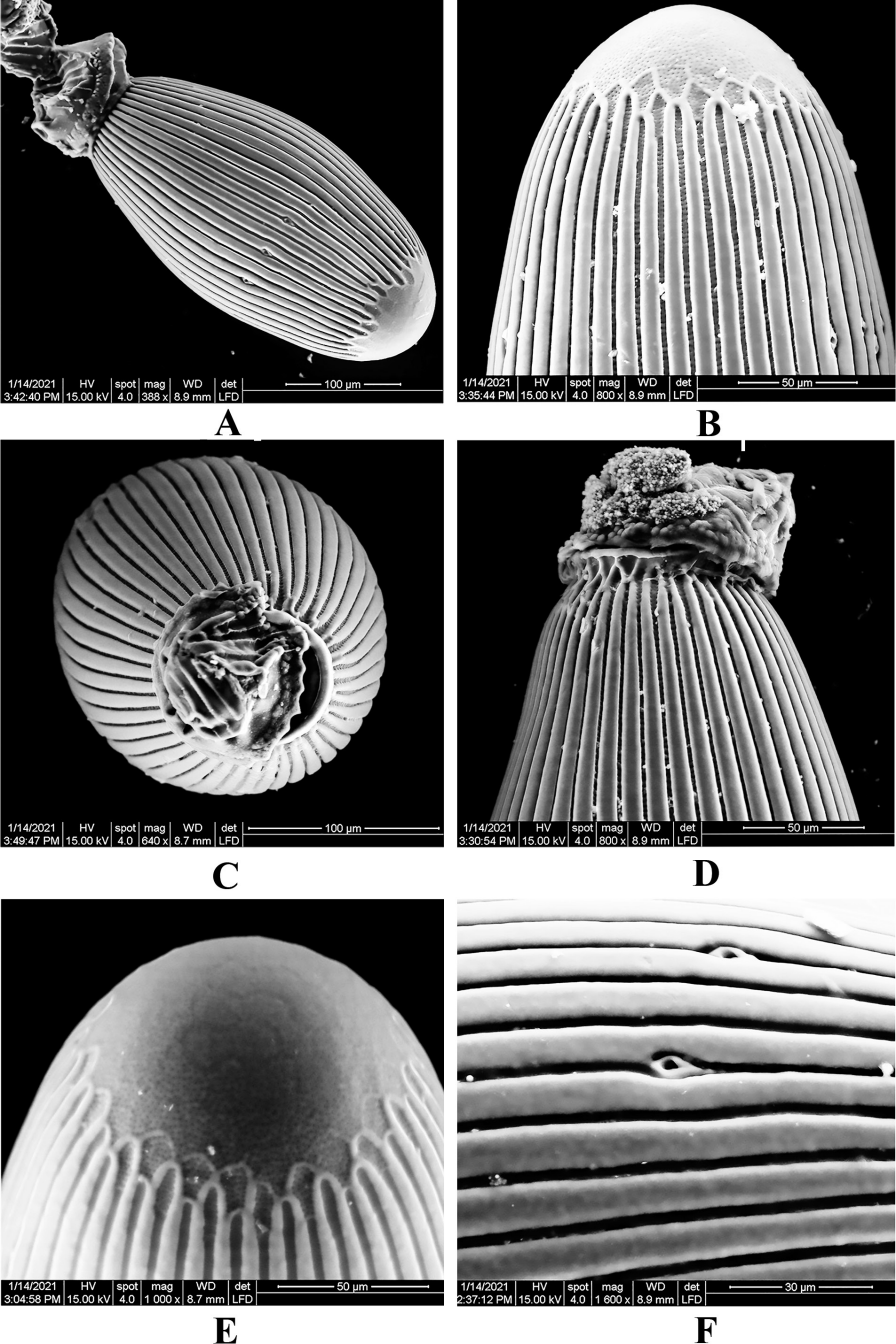
*Neoperlahajeki* sp. nov. (egg) **A** egg **B** lid **C** collar **D** collar and lid **E** FCIs and lid **F** micropyles.

***Egg*** (Fig. 6). Chorion length 346–348 μm, width 173–177 μm. Micropyles 3 with rims, placed ca 1/3 length near opercullum, each located between striae (Fig. 6A, F). Collar not distinctly stalked, but slightly constricted at base; width ca 79.5 μm at collar (Fig. 6D). Collar short, irregular in shape (Fig. 6D). Rim slightly flanged, margin irregularly scalloped. Chorion with striate on lid and collar. FCIs on lid distinct; cells with thin, smooth walls and floors punctuated (Fig. 6B, E).

#### Etymology.

The species is named after Dr Jiri Hájek for collecting the specimens.

#### Distribution.

China (Yunnan).

#### Ecology.

See ecology of *Neoperlagaoligongshana* sp. nov.

#### Remarks.

The new species is a member of the *N.clymene* group. Color pattern, pronotum, and male genital features are similar to *Neoperlaorissa* Stark & Sivec, 2015 from India. The new species can be easily separated from *N.orissa* by the projection of tergum 7 and detail of the aedeagal sac armature. In *N.hajeki*, the projection of tergum 7 is pointed in dorsal aspect, and the dorsal spines of the aedeagal sac are triangularly arranged. In *N.orissa*, the slender and median projection of tergum 7 appears truncate in dorsal aspect, and the spines of the sac are arranged in several close-set rows.

### ﻿ Neoperla lihuae

Taxon classificationAnimaliaPlecopteraPerlidae

Li & Murányi, 2014

3AB8503B-A9A8-5A36-87B5-D5FB763F81B7

Neoperla lihuae Li and Murányi 2014: 2 (original description).

#### Material examined.

1 male and 3 females (NMP), 1 male and 1 female (HIST), China: Yunnan Province, Yingjiang County, Dehong Dai and Jingpo Autonomous Prefecture, Tongbiguan Town, at light in village near river, 24–26.VI.2016, 24°36.7'N, 97°39.4'E, 1340 m, leg. J. Hájek & J. Růžička.

#### Distribution.

China: Yunnan.

#### Ecology.

Tongbiguan Town is located in southwestern Yingjiang County, and it also belongs to the Gaoligongshan region. At the same locality, accompanying stoneflies were *Neoperlahubleyi* Stark & Sivec, 2008 and one unidentified female *Neoperla* sp.

#### Remarks.

*Neoperlalihuae* Li & Murányi, 2014 was originally described by Li et al. (2014a) from Xishuangbanna in Yunnan Province. Our specimens from Yingjiang County agree well with original description of the head pattern and terminalia, the aedeagal tube and sac.

### ﻿ Neoperla hubleyi

Taxon classificationAnimaliaPlecopteraPerlidae

Stark & Sivec, 2008

DB8DE4AF-5138-5352-A311-964E0C49F73B

Neoperla hubleyi Stark and Sivec 2008: 30 (original description); Mo et al. 2020: 521 (new record for Guangxi Zhuang Autonomous Region).

#### Material examined.

1 male (HIST), China: Yunnan Province, Dehong Dai and Jingpo Autonomous Prefecture, Yingjiang County, Tongbiguan Town, at light in village near river, 24–26.VI.2016, 24°36.7'N, 97°39.4'E, 1340 m, leg. J. Hájek & J. Růžička.

#### Distribution.

China: Yunnan and Guangxi Zhuang Autonomous Region; Vietnam.

#### Ecology.

See ecology of *Neoperlalihuae* Li & Murányi, 2014.

#### Remarks.

Stark and Sivec (2008) originally described this species from Vietnam, and Mo et al. (2020a) described a new record in Guangxi Zhuang Autonomous Region. The terminalia and aedeagus of our specimen fit the original description of Stark and Sivec (2008).

##### ﻿Concluding remarks

*Neoperla* was divided into the *Neoperlaclymene* group and the *Neoperlamontivaga* group by Zwick (1983). The *N.clymene* species group currently includes more than 150 species, and the *N.montivaga* species group includes over 123 species worldwide (Huo et al. 2021; Mo et al. 2021a).

**Figure 7.**
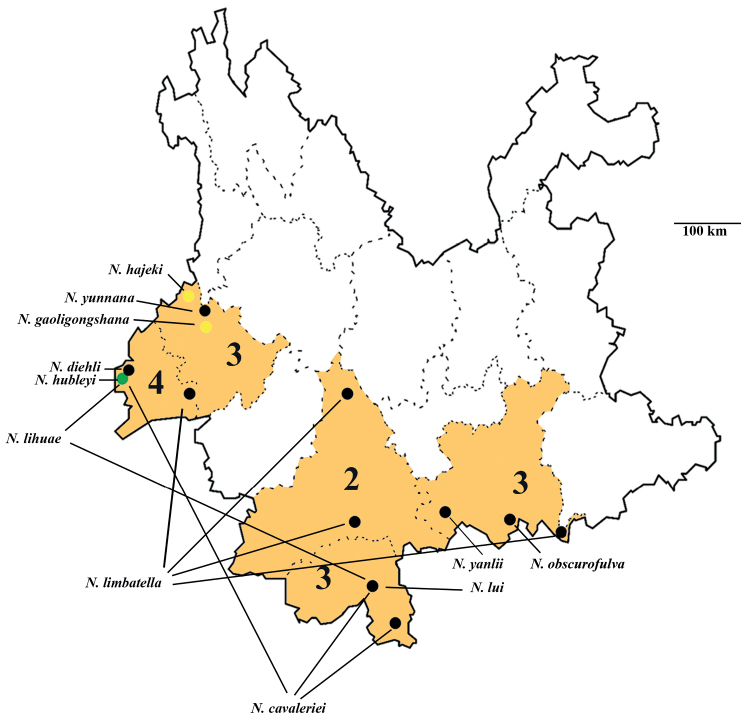
Distribution of *Neoperla* Needham, 1905 in cities of Yunnan. Yellow dots indicate location of the two new species, green dot indicates two new records.

So far 11 species of *Neoeprla* have been recorded from Yunnan Province, including the two new species and one species newly recorded in this paper. Among the six endemic species of *Neoperla*, including the two new species listed below—*N.lihuae*, *N.yanlii*, *N.obscurofulva*, *N.yunnana*, *N. hájeki*, and *N.gaoligongshana*—three of them are distributed in Baoshan City. Five species are widely distributed: *N.cavaleriei*, *N.diehli*, *N.hubleyi*, *N.limbatella*, and *N.lui*. *Neoperlabinodosa* Wu, 1973 was transferred to the genus *Phanoperla* and is placed as a synonym of *P.pallipennis* Banks, 1938 by Mo et al. (2021c). Most species are distributed in western and southeastern Yunnan Province and central and northern Yunnan still needs to be surveyed (Fig. 7).

**Figure 8.**
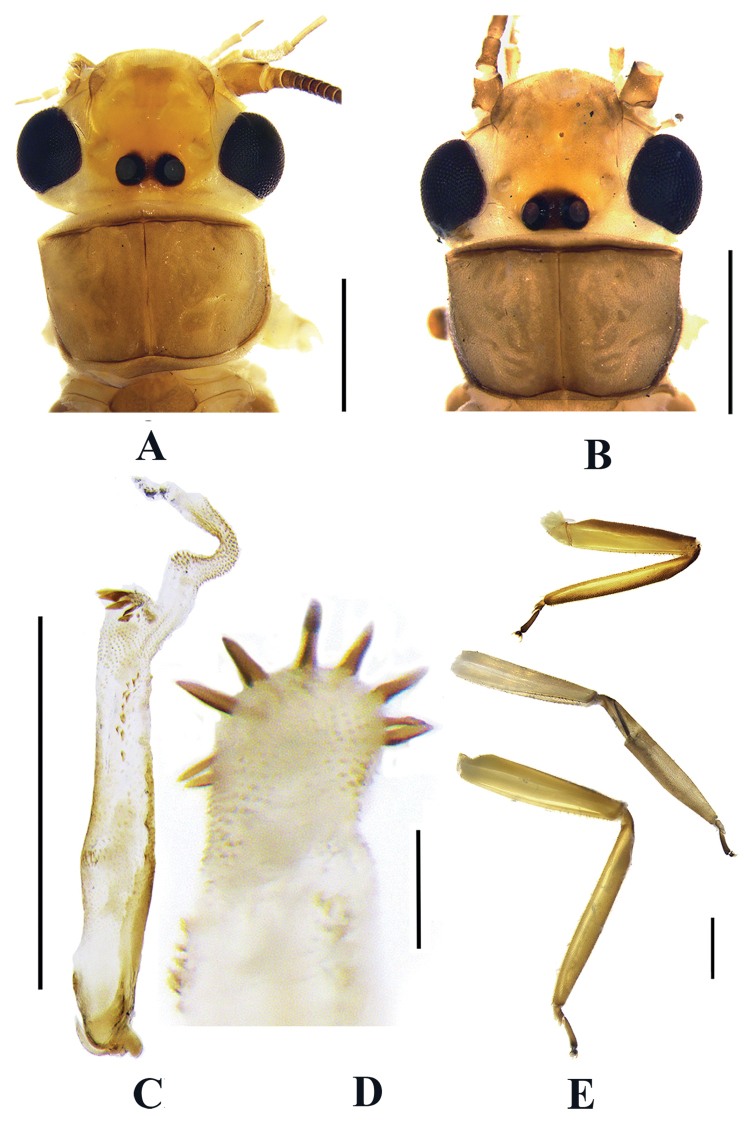
*Neoperlawuzhishana* Chen & Du, 2016 (**A, C–E** male **B** female), all from Yinggeling, Hainan Province **A** head and pronotum, dorsal view **B** head and pronotum, dorsal view **C** Aedeagus, lateral view **D** Large spines of aedeagal sac, ventral view **E** legs, dorsal view. Scale bars: 1 mm (**A–C, E**); 0.1 mm (**D**).

## Supplementary Material

XML Treatment for Neoperla gaoligongshana

XML Treatment for Neoperla hajeki

XML Treatment for Neoperla lihuae

XML Treatment for Neoperla hubleyi
